# Event-Related Potentials During Decision-Making in a Mixed-Strategy Game

**DOI:** 10.3389/fnins.2021.552750

**Published:** 2021-03-19

**Authors:** Fang-Yu Chang, Winnugroho Wiratman, Yoshikazu Ugawa, Shunsuke Kobayashi

**Affiliations:** ^1^Department of Neurology, School of Medicine, Fukushima Medical University, Fukushima, Japan; ^2^Department of Neurology, Faculty of Medicine, Universitas Indonesia, Cipto Mangunkusumo Hospital, Jakarta, Indonesia; ^3^Department of Human Neurophysiology, Fukushima Medical University, Fukushima, Japan; ^4^Department of Neurology, Takeda General Hospital, Fukushima, Japan; ^5^Department of Neurology, Teikyo University, Tokyo, Japan

**Keywords:** levodopa, feedback, readiness potential, game theory, Parkinson’s disease, high-density EEG, prefrontal cortex, executive function

## Abstract

The decisions we make are sometimes influenced by interactions with other agents. Previous studies have suggested that the prefrontal cortex plays an important role in decision-making and that the dopamine system underlies processes of motivation, motor preparation, and reinforcement learning. However, the physiological mechanisms underlying how the prefrontal cortex and the dopaminergic system are involved in decision-making remain largely unclear. The present study aimed to determine how decision strategies influence event-related potentials (ERPs). We also tested the effect of levodopa, a dopamine precursor, on decision-making and ERPs in a randomized double-blind placebo-controlled investigation. The subjects performed a matching-pennies task against an opposing virtual computer player by choosing between right and left targets while their ERPs were recorded. According to the rules of the matching-pennies task, the subject won the trial when they chose the same side as the opponent, and lost otherwise. We set three different task rules: (1) with the alternation (ALT) rule, the computer opponent made alternating choices of right and left in sequential trials; (2) with the random (RAND) rule, the opponent randomly chose between right and left; and (3) with the GAME rule, the opponent analyzed the subject’s past choices to predict the subject’s next choice, and then chose the opposite side. A sustained medial ERP became more negative toward the time of the subject’s target choice. A biphasic potential appeared when the opponent’s choice was revealed after the subject’s response. The ERPs around the subject’s choice were greater in RAND and GAME than in ALT, and the negative peak was enhanced by levodopa. In addition to these medial ERPs, we observed lateral frontal ERPs tuned to the choice direction. The signals emerged around the choice period selectively in RAND and GAME when levodopa was administered. These results suggest that decision processes are modulated by the dopamine system when a complex and strategic decision is required, which may reflect decision updating with dopaminergic prediction error signals.

## Introduction

Interactive decision-making is fundamental to our social activity. However, it is elusive, in that it may change dynamically depending on social contexts and knowledge about the intentions of others. For example, playing the rock-paper-scissors game involves complex interactive processes including decision variables such as likelihoods, priors, and values. The game theory provides a mathematical framework for the strategic interactions among decision-makers. In the game theory, a player is said to use a mixed strategy whenever they choose to randomize over the set of available actions (Dixit et al., 2009). Mixed-strategy games have been used for neurophysiological research on decision-making and identified neural signals related to the previous choices, previous rewards, expected reward value, and decision switching of subjects in the medial prefrontal cortex and other brain areas (Barraclough et al., 2004; Dorris and Glimcher, 2004; Seo and Lee, 2007, 2008; Seo et al., 2009; Thevarajah et al., 2009; Donahue et al., 2013). These neural signals may reflect strategic updating of decision variables based on past history.

There are several brain regions involved in decision-making, among which the dopamine system and prefrontal cortex appear to play pivotal roles, particularly when a decision involves a strategic interaction (Lee and Seo, 2016). There is converging evidence that the dopamine system is essential for adapting behavior based on previous outcomes, such as dopamine neurons emitting teaching signals that guide reinforcement learning (Schultz et al., 1997), dopamine activity reflecting the upcoming choices and actions of subjects (Morris et al., 2006; Yun et al., 2020), and the economic decisions of Parkinson’s disease (PD) patients being influenced by dopaminergic treatment (Kobayashi et al., 2019). Decision processes can be decomposed into multiple tasks, including integration of external information, outcome estimation, and movement preparation. The primate prefrontal cortex has been shown to be involved in each of these cognitive tasks, and is thus thought to be one of the main processors involved in decision-making to guide goal-directed behavior (Kim and Shadlen, 1999; Krawczyk, 2002; Matsumoto et al., 2003). Anatomically, dopamine neurons project to the prefrontal cortex *via* the mesocortical pathway. Given these anatomical and physiological backgrounds, it is tempting to speculate that decision substrates are formed in the prefrontal cortex under the influence of dopaminergic input that carries reinforcement signals and motivational drive. However, we are not aware of any previous study that has directly examined dopaminergic influences on behavior and electrophysiological activities during an interactive game.

We aimed to determine how dopamine influences event-related potentials (ERPs) while healthy subjects played a matching-pennies task, which is a type of mixed-strategy zero-sum game. ERPs are voltage changes in electroencephalography (EEG) recordings that occur before, during, or after physical or mental events (Picton et al., 2000). Neural correlates of decision-making in humans have been studied mainly using functional magnetic resonance imaging (fMRI). However, decision processes are dynamic and instantaneous, possibly occurring in the subsecond range, and so fMRI data with a low temporal resolution must be interpreted with caution. Recording ERPs is well suited to studying decision processes since it yields non-invasive data on cortical activity with excellent temporal resolution. Much of the ERP research on decision-making has focused on postdecision activity, such as error-related negativity (Falkenstein et al., 1991) and the central–parietal P3 component (Polich, 2007). We looked for ERP correlates of strategic decision-making, focusing on prefrontal activity and dopaminergic influences.

According to the rules of the matching-pennies task, the subject won the trial when they chose the same side as the opponent, and lost otherwise. We set three different task rules: (1) with the alternation (ALT) rule, the computer opponent made alternating choices of right and left in sequential trials; (2) with the RAND rule, the opponent randomly chose between right and left; and (3) with the GAME rule, the opponent analyzed the subject’s past choices in order to predict the subject’s next choice, and then chose the opposite side. Therefore, the subject had to make choice patterns as random as possible in order to reduce the likelihood of the computer opponent winning. We hypothesized that the prefrontal cortex would exhibit greater activation when decisions were based on complex priors rather than the simple alternation rule: in order words, when decision-making is based on intensive computation of complex priors and outcome prediction, dopaminergic input to the prefrontal cortex plays a critical role. We therefore tested whether the administration of levodopa, a precursor of dopamine, enhances decision-related ERPs.

## Materials and Methods

### Participants

Eighteen right-handed healthy adults (12 males; age 46.2 ± 12.8 years, mean ± *SD*) were recruited. One male subject was excluded because he withdrew from the experiment after the first session. Fukushima Medical University Ethical Committee approved the experimental design of the study, and informed consent was obtained from all subjects.

### Procedures

Each subject participated in recording sessions twice, separated by an interval of at least 1 week. The subjects took either a placebo or 100 mg of levodopa 45 min before the start of each recording. Drug treatment was double-blinded and randomized.

**FIGURE 1:**
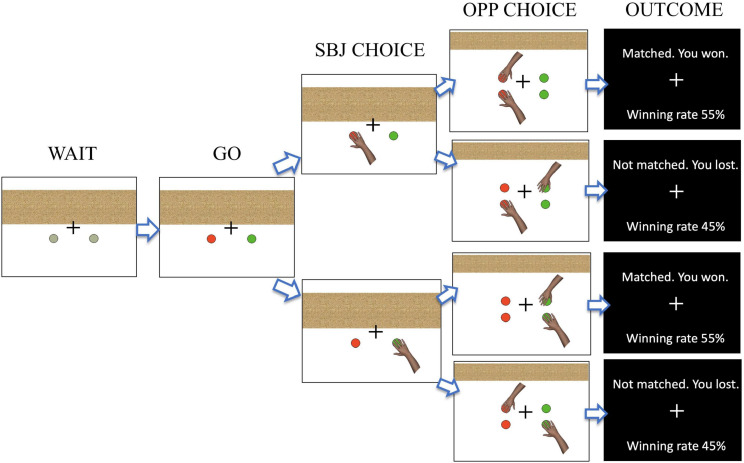
Illustration of the task flow in the matching-pennies task. The task started with the presentation of two gray circles (WAIT). A color change of the two circles signaled the subject to make a choice response (GO), with the subject having to choose either the left or right target (SBJ CHOICE). After a 1,000 ms delay, the choice made by the computer player was displayed (OPP CHOICE). After 700 ms, the outcome was shown (OUTCOME).

During EEG recordings, the subject was seated 70 cm from a computer monitor (60 cm × 30 cm) and played a computerized version of the matching-pennies task (Figure 1). At the beginning of each trial, two gray circles were displayed at the bottom of the monitor and the subject put the index and middle fingers of their right hand on the two designated keys on the keyboard (during the WAIT period). After 800 ms, the gray circles changed color, which signaled the subject to press either one of the two keys (the GO period). Immediately after they had made their choice, a hand illustration was displayed below the chosen side of the circles (designated as SBJ CHOICE). After 1,000 ms, the choice of the computer player was revealed by a hand illustration shown on the left or right side at the top of the monitor (OPP CHOICE). The subject won the trial if the side of their choice matched that of the computer player, and otherwise, the subject lost (Table 1). After 700 ms, the outcome of each trial was displayed on the monitor together with the total winning rate (OUTCOME). The subject was encouraged to win as much as possible. The trial was aborted if the subject responded during the WAIT period or did not respond within 5 s after the GO signal.

**TABLE 1:** Payoff matrix of the subject and the computer player for the choice of left vs. right key presses (subject, computer).

		Computer player’s choice	
		Left	Right
Subject’s choice	Left	(Win, lose)	(Lose, win)
	Right	(Lose, win)	(Win, lose)

The following algorithms were implemented for the computer player: Each subject performed the matching-pennies task with three different rules. For the ALT rule, in which the computer player chose the right or left target alternatively, the subject could easily predict the opponent’s choice. However, the subject could not predict the opponent’s choice for the RAND rule, since the computer player chose the right or left target randomly with equal probability. The GAME rule was modified from a previous study (Barraclough et al., 2004), in which the computer could exploit systematic bias in the subject’s choice in the recent past to maximize the winning rates of the computer player. The computer saved the entire history of the subject’s choices in a given session and used the information to predict the subject’s next choice by testing a set of hypotheses. Multiple conditional probabilities were estimated to predict the subject’s choice, and the computer biased its selection according to these probabilities.

*P*_(A,_ *_*N*_*_)_ is the conditional probability of the subject choosing A (right or left) given that they have chosen A in the *N*th previous trials. For example, if the subject chose the right target with a probability of 80% in the recent past (i.e., *P*_(left, 1__)_ = 0.8), the probability of the computer selecting the left target would be 80%. The subject’s choice may be conditional not only on their past choice but also on the game outcome (win vs. lose). *P*_w__in__(A,_ *_*N*_*_)_ and *P*_lose__(A,_ *_*N*_*_)_ are the conditional probabilities of the subject choosing A given that they chose A in the *N*^th^ previous trials and winning or losing the trial, respectively, for example, *P*_win__(A, 1__)_ = 1 means a win-stay strategy and *P*_lose(A, 1__)_ = 0 means a lose-switch strategy. Among these conditional probabilities computed for *N* = 1–4, the probability with the highest value (*P*_max_) was taken to bias the choice of the computer player to the opposite side at the same probability:

(1) $$P_{\text{max}}=\text{max}\,\left(P_{\left(A,N\right)},P_{\text{win}\,\left(A,N\right)},P_{\text{lose}\,\left(A,N\right)}\right)\mathrm{for}\,A=\mathrm{left},\mathrm{right}\,\mathrm{and}\,N=1,2,3,4.$$

The next choice of the computer player was opposite to the subject’s choice with a probability of *P*_max_.

All of the subjects played 450 trials, comprising six sessions with three different rules and 75 trials/session. Six blocks of trials were run in a fixed order (ALT–RAND–GAME–ALT–RAND–GAME) and there was a short break between the trial blocks. At the beginning of the experiment, we first provide general instructions about the task sequence and procedure of the matching-pennies task, including showing example screenshots. We then explained the three different rules and allowed 15 practice trials for each rule.

The following instructions were given for each task rule:

ALT rule: “In this session, the computer opponent will simply alternate its response between right and left. So, you can easily win by alternating your response to choose the side that the opponent will choose.”
RAND rule: “In this session, the computer opponent will randomly choose between right and left. So, its response is unpredictable. You will win or lose by chance.”
GAME rule: “In this session, the computer opponent will analyze your past choices in order to predict your next choice. For example, if you chose left repeatedly, the computer opponent would choose right to win. Therefore, you have to try to make unpredictable selection patterns to reduce the likelihood of the computer opponent winning. You are encouraged to win as much as possible.”

The three rules were applied in a fixed order to make it as easy for the subjects to understand which rule they were performing for a particular task. The sequence of ALT–RAND–GAME was repeated twice to avoid the possible order effect, training effect, and effects of changes in the recording conditions such as in the electrode impedances. We explicitly provided instructions about the relevant rule to the subjects before a new session began. We also instructed them to look at the cross mark at the center of the monitor to avoid eye movements. The behavioral task was controlled by MATLAB (version R2014a, The MathWorks, Natick, Massachusetts, United States) with Cogent 2000 and the Psychophysics Toolbox (Brainard, 1997) running on a Windows computer connected to a Macintosh computer that sampled the EEG data.

### Data Acquisition and Analysis

Seventeen subjects completed the entire experiment, and all of their behavioral data were included in the behavioral analysis. We analyzed the winning rates of the subjects for each rule. We evaluated how much the choice of a subject was influenced by their own choices in the past by using the log likelihood ratio (LR):

(2) $${\text{LR}}_{\text{self}}\left(N\right)=\text{log}[\left\{P\left({\text{rt}}_{\text{self}},{\text{rt}}_{\text{self}}\right)+P\left({\text{lt}}_{\text{self}},{\text{lt}}_{\text{self}}\right)\right\}/\{P\left({\text{rt}}_{\text{self}},{\text{lt}}_{\text{self}}\right)+P\left({\text{lt}}_{\text{self}},{\text{rt}}_{\text{self}}\right)\}]$$

where LR_sel__f_(*N*) is the LR of choosing the same side in the current trial as in the *N*^th^ previous trials, rt and lt correspond to pressing the right- and left-side keys as the decision, respectively, and *P*(A_self_, B_self_) is the condition probability of the subject choosing A in the current trial and choosing B in the *N*^th^ previous trials. LR_sel__f_(*N*) = 0 indicates that past choices did not influence the present choice. If the subjects tended to choose the same side as or the opposite side to the choice in the *N*^th^ previous trials, LR_sel__f_(*N*) would be positive or negative, respectively.

The subject’s current choice could also be influenced by the opponent’s choice in the previous trials. The influence from the past choices of the computer player was evaluated in an analogous manner:

(3) $${\text{LR}}_{\text{pc}}\left(N\right)=\text{log}[\left\{P\left({\text{rt}}_{\text{self}},{\text{rt}}_{\text{pc}}\right)+P\left({\text{lt}}_{\text{self}},{\text{lt}}_{\text{pc}}\right)\right\}/\{P\left({\text{rt}}_{\text{self}},{\text{lt}}_{\text{pc}}\right)+P\left({\text{lt}}_{\text{self}},{\text{rt}}_{\text{pc}}\right)\}]$$

where LR_pc_(*N*) is the LR of choosing the same side in the current trial as the opponent’s choice in *N* previous trials, and *P*(A_self_, B_pc_) is the conditional probability of the subject choosing A in the current trial when the computer player chose B in the *N*^th^ previous trials. Logistic regression analysis was used to identify how the past choices influenced the current choice (see Supplementary Material 1 for details).

To measure the randomness of choosing between right and left, we calculated the permutation entropy of the numerical sequences of the chosen target using the MATLAB function “pec.m” (Bandt and Pompe, 2002; Ouyang, 2020).

EEG data were recorded from 129 scalp locations at a sampling rate of 1,000 Hz with a 24-bit resolution (Geodesic EEG System 400, Electrical Geodesic, Eugene, Oregon, United States). EEG data analysis was performed using EEGLAB (version 14.1.1) (Delorme and Makeig, 2004) and ERPLAB (version 6.1.4) (Lopez-Calderon and Luck, 2014). The data were downsampled to 250 Hz and bandpass filtered between 0.01 and 30 Hz, with a notch filter at 50 Hz. Noisy channels were removed, and the removed channels were interpolated. The sampled data were re-referenced to the average of all electrodes. Independent-components analysis and an automatic EEG artifact detector (Mognon et al., 2011) were used to correct for artifacts such as blinks and eye movements.

ERPs were analyzed in three periods. The first period (period I) was from the onset of the WAIT cue until the onset of the GO cue (800 ms), which corresponds to the period when the subject waited for the GO stimulus to appear on the monitor. The second period (period II) was from -800 to 0 ms from the subject’s choice response (SBJ CHOICE, 800 ms). The third period (period III) was from the disclosure of the opponent’s choice (OPP CHOICE) until the onset of the feedback cue (OUTCOME, 700 ms). The activity during the intertrial interval was used for baseline correction. We excluded EEG data with a large proportion of noise contamination (more than 25% of trials rejected by ERPLAB).

For periods I and II, the period-mean amplitude was defined as the amplitudes of ERPs averaged over the entire period. For periods I to III, the negative peak amplitude was defined as the minimum value of the ERP during each period. For period III, the positive peak amplitude was defined as the maximum value of the ERP during the period.

Initially, there were 17 subjects. Data from seven subjects were excluded because the number of trials was insufficient after artifact removal. Therefore, data from the remaining 10 subjects were analyzed. The numbers of trials averaged for the ERP analysis are summarized in Table 2.

**TABLE 2:** Numbers of trials used to obtain event-related potentials (ERPs).

	Rule	Period I	Periods II and III
Placebo	ALT	134.3 (115–150)	130.1 (100–150)
	RAND	132.7 (119–148)	128.6 (101–148)
	GAME	132.8 (106–149)	133.1 (107–149)
Levodopa	ALT	130.1 (97–148)	124.4 (83–148)
	RAND	132.7 (111–150)	127.8 (95–150)
	GAME	133.9 (112–149)	130.9 (109–150)

*EEG noise contamination was removed separately for period I and periods II and III, and hence, the number of trials differed across periods. Data are mean (range) values for 10 subjects.*

To quantify the selectivity of the ERP signals to the direction of the subject choice, we calculated the mean ERP difference between choosing the right and left targets:

(4) $${\text{ERP}}_{\left(\mathrm{rt}-\mathrm{lt}\right)}={\text{ERP}}_{\text{mean}}\,\left(\text{rt}\right)-{\text{ERP}}_{\text{mean}}\,\left(\mathrm{lt}\right)$$

where ERP_mean_(rt) and ERP_mean_(rt) are the ERPs averaged for trials in which the subject chose the right and left, respectively. To quantify the ERP selectivity for behavioral outcomes, we calculated the mean ERP difference between winning and losing trials:

(5) $${\text{ERP}}_{\left(\mathrm{win}-\mathrm{lose}\right)}={\text{ERP}}_{\text{mean}}\,\left(\mathrm{win}\right)-{\text{ERP}}_{\text{mean}}\,\left(\mathrm{lose}\right)$$

where ERP_mean_(win) and ERP_mean_(lose) are the ERPs averaged for trials in which the subject won and lost, respectively.

To examine whether ERPs were influenced by the choice history, we examined the effect of previous choices from the following aspects:

1. The influence of the previous choice was examined by calculating the mean ERP difference between trials in which subjects chose the right and left targets in the preceding trial (Supplementary Material 2).
2. The influence of the outcome in the previous trial was examined by calculating ERP_(win__–__lose)_ based on the outcome in the preceding trial:

(6) $${\text{ERP}}_{\mathrm{prev}\,\left(\mathrm{win}-\mathrm{lose}\right)}={\text{ERP}}_{\text{mean}}\,\left(\mathrm{won}\,\mathrm{in}\,\mathrm{previous}\,\mathrm{trial}\right)-{\text{ERP}}_{\text{mean}}\,\left(\mathrm{lost}\,\mathrm{in}\,\mathrm{previous}\,\mathrm{trial}\right)$$

where ERP_mean_(won in previous trial) and ERP_mean_(lost in previous trial) are the ERPs averaged for trials just after winning and losing, respectively.
3. Whether subjects changed the choice direction from the preceding trial (switch) or chose the same direction as before (stay) was examined by calculating the mean ERP difference between switch and stay trials:

(7) $${\text{ERP}}_{\left(\mathrm{switch}-\mathrm{stay}\right)}={\text{ERP}}_{\text{mean}}\,\left(\mathrm{switch}\right)-{\text{ERP}}_{\text{mean}}\,\left(\mathrm{stay}\right)$$

where ERP_mean_(switch) and ERP_mean_(stay) are the ERPs averaged for trials in which the subject had switched from or stayed with the previous trials, respectively.

### Statistical Analysis

#### Behavioral Data

Average winning rates were subjected to two-way repeated-measures ANOVA for rule (RAND and GAME) × drug (levodopa and placebo). Behavioral data in the ALT condition were not included because the subjects won almost 100% of those trials. Because reaction time data were not normally distributed, the data were inverse Gaussian transformed. Data on winning rates, reaction time, and permutation entropy were analyzed using two-way repeated-measures ANOVA for rule × drug. When the permutation entropy was tested by ANOVA, data in the ALT condition were not included because behavioral responses were simple alternations and their permutation entropy was fixed at 0.69.

The significance criterion was set at *p* < 0.05 and the effect size was quantified using partial eta-squared (η_p_^2^). We tested whether LR_sel__f_(*N*) and LR_pc_(*N*) differed significantly from 0 using a two-tailed Student’s *t*-test with Bonferroni correction.

#### Electrophysiological Data

The effects of drug and rule on the ERPs were tested using two-way repeated-measures ANOVA for rule (ALT, RAND, and GAME) × drug (levodopa and placebo). The significance criterion was set at *p* < 0.05 with multiple comparisons corrected using Tukey HSD. We also tested ERPs using three-way ANOVA for rule (ALT, RAND, and GAME) × drug (levodopa and placebo) × outcome (win and lose) and three-way ANOVA for rule (ALT, RAND, and GAME) × drug (levodopa and placebo) × choice (switch and stay). The significance criterion was set at *p* < 0.05. The analyses were conducted using the MATLAB Statistics toolbox and SPSS statistical software (version 23, IBM, Armonk, New York, United States).

## Results

### Behavioral Results

**FIGURE 2:**
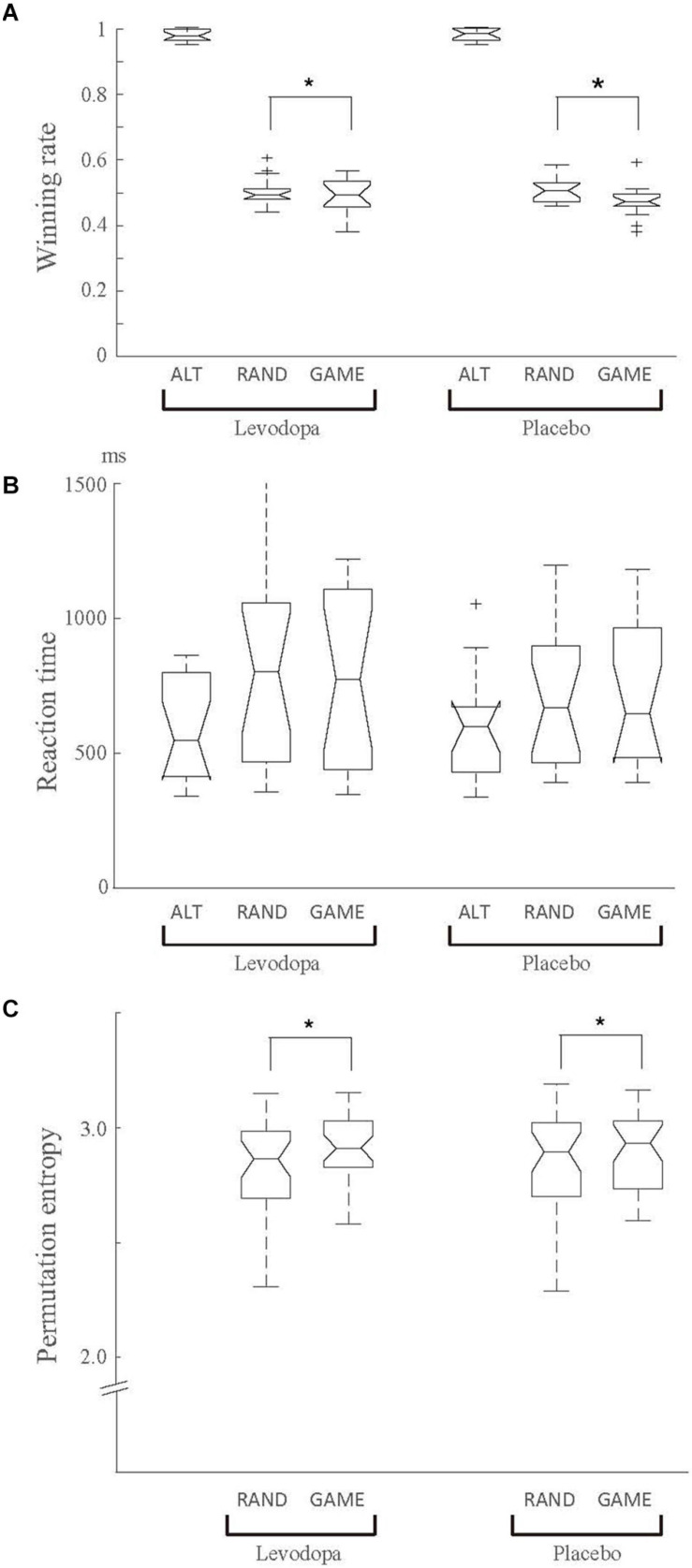
Behavioral results for the winning rate **(A)**, behavioral reaction time **(B)**, and permutation entropy of the choice sequence **(C)**. Data were averaged for each task rule and for each drug condition (levodopa or placebo) across 17 subjects. **(A)** There was a significant main effect of rule in two-way repeated-measures ANOVA (*p* < 0.05), indicating that the winning rate was higher in RAND than in GAME. There was no significant main effect of drug or an interaction effect (*p* > 0.05). **(B)** The choice reaction time did not change significantly with the rule or levodopa treatment. **(C)** The permutation entropy measured randomness of the choice sequence. Permutation entropy in ALT was fixed at 0.69 (not plotted). Entropy was slightly but significantly higher in GAME than in RAND (drug main effect, *p* = 0.02 in two-way repeated-measures ANOVA). In each box plot, the central line indicates the median, the bottom and top edges of the box indicate the 25th and 75th percentiles, respectively, and the whiskers extend to the extreme data points that were not considered outliers; the outliers are indicated separated by the “+” symbols. *p < 0.05 for the main effect in ANOVA.

Figure 2A shows the winning rates for the three task rules in the levodopa and placebo groups. The behavioral performance in ALT sessions was nearly perfect, as expected, reflecting how easy it was to predict the opponent’s choice. The behavioral performance in RAND and GAME was close to the chance level, but significantly worse for GAME than for RAND, as supported by a significant main effect of rule in two-way repeated-measures ANOVA for rule × drug on winning rate [*F*_(__1, 16)_ = 8.515, *p* = 0.01, η_p_^2^ = 0.068]. There was no significant main effect of drug or an interaction effect. The choice reaction time was 623.0 ± 301.4 ms overall, and it did not change significantly with the rules or levodopa treatment (two-way repeated-measures ANOVA, *p* > 0.05; Figure 2B).

In GAME sessions, the subjects could play better by choosing targets independently in each trial, because any choice bias could have been exploited by the computer. Figure 2C shows the permutation entropy (a measure of response randomness) in RAND and GAME. The behavioral response in ALT was a fixed sequence, and so its permutation entropy was fixed at 0.69. The permutation entropy was significantly higher in GAME than in RAND when applying two-way repeated-measures ANOVA for rule × drug [*F*_(__1, 16__)_ = 6.374, *p* = 0.02, η_p_^2^ = 0.056]. These results indicate that the subjects made more random choices in GAME (Figure 2C).

**FIGURE 3:**
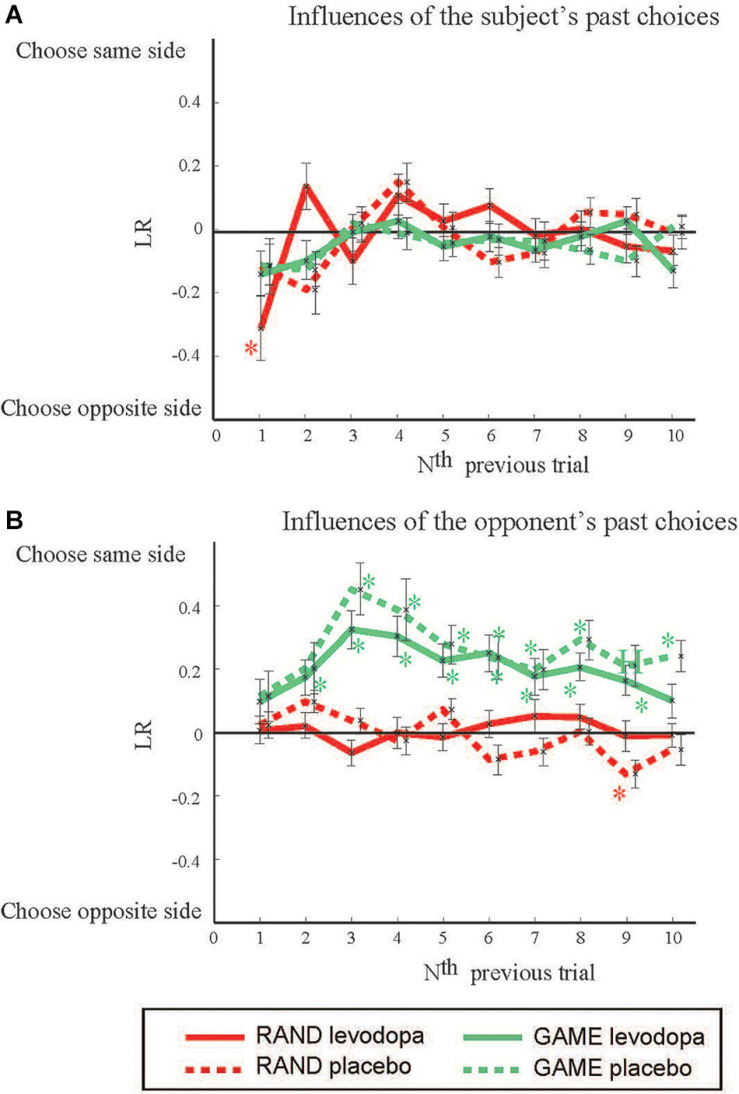
The effects of previous choices on decision-making. The figure shows how past choices influenced the present choice by plotting the LR vs. how long ago the previous trial occurred. Positive and negative LRs mean that the subject tended to choose the same side as and the opposite side to that in the previous trials, respectively. **(A)** Influences from the subject’s own past choices. **(B)** Influences from the opposing player’s past choices. Data are mean and *SD* values. *p < 0.05 (Student’s *t*-test) for deviation from 0.

We evaluated the degree to which the choices made by subjects were influenced by their own choices in the past by using the LR method (cf. Supplementary Material 1 for the results by logistic regression analysis). LR_sel__f_(*N*) expresses the influences from the past choices of the subject in the *N*^th^ previous trials (Figure 3A). LR_sel__f_(1) was significantly negative in RAND sessions with levodopa [*t*(35) = 2.968, *p* = 0.05, Student’s *t*-test with Bonferroni correction], indicating that subjects tended to choose the target opposite to the one they chose in a previous trial. There was no significant past influence in GAME sessions. In a similar way, we analyzed the influences of the opponent’s past choices (Figure 3B). LR_pc_(*N*) was significantly positive in GAME sessions for *N* = 2–9 with levodopa [*t*(31) = 3.14, 5.49, 4.83, 4.38, 4.23, 3.11, 4.62, and 3.47, respectively; *p* < 0.005] and for *N* = 3 to 10 without levodopa [*t*(33) = 5.48, 3.90, 4.50, 3.42, 3.10, 4.67, 3.22, and 4.88, respectively, *p* < 0.05]. These results indicate that the subjects tended to choose the target on the same side as the target chosen by the computer player in the past.

### Electrophysiological Results

**FIGURE 4:**
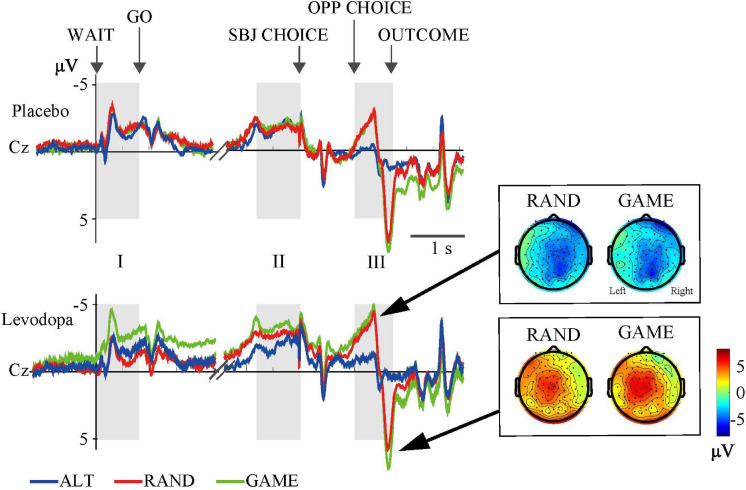
Grand-averaged waveforms at the Cz electrode for the three rules. Event-related potentials (ERPs) were averaged from 10 subjects in three task conditions. Gray areas (I, II, and III) indicate time windows of the ERP analyses (cf. Table 3). Topographical maps show the distributions of negative (top) and positive (bottom) peaks during period III.

**FIGURE 5:**
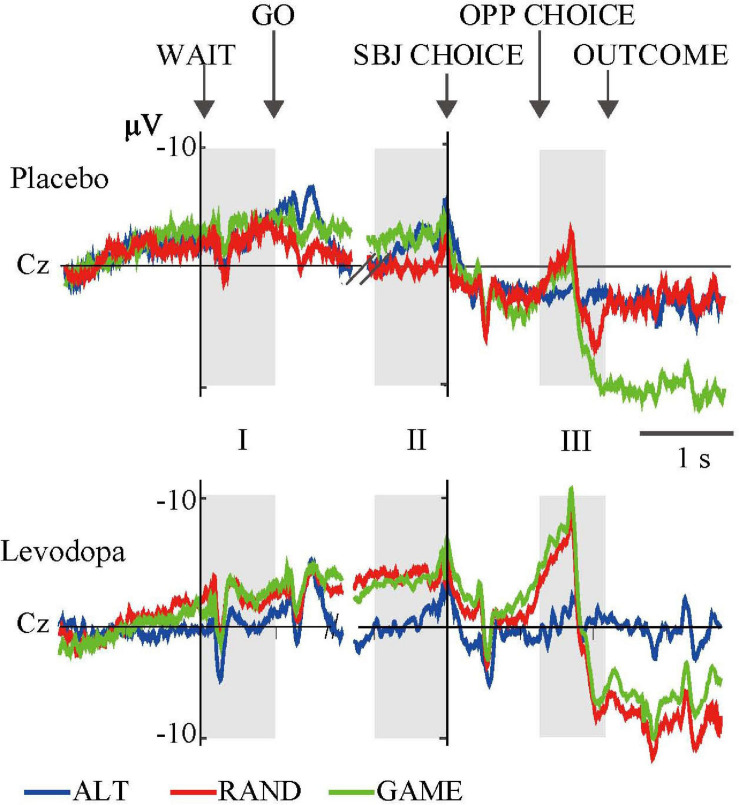
Grand-averaged waveforms of a representative single subject recorded at the Cz electrode for the three rules. The format is the same as in Figure 4.

We averaged EEG activities from 10 subjects and aligned them to the trial start (WAIT) and the subject’s choice response (SBJ CHOICE). The activity recorded at the Cz electrode is plotted separately for the three different task rules with and without levodopa in Figure 4. The ERP profile of a representative single subject recorded at the Cz electrode is shown in Figure 5. Period I covers the movement preparation period from the WAIT cue until the GO cue (800 ms). After the phasic response to the WAIT cue, we observed sustained negativity particularly during GAME and RAND sessions with levodopa between periods I and II, which returned to the baseline after the subjects made the key-press response (SBJ CHOICE). During period III, when the opposing player’s choice was displayed on the monitor, the negative potential increased again, which resulted in an abrupt deflection toward positivity and a return to the baseline after the outcome information was displayed on the monitor. For the RAND and GAME rules, the negative and positive peaks were distributed around the vertex electrodes during period III (topographical maps in Figure 4).

**FIGURE 6:**
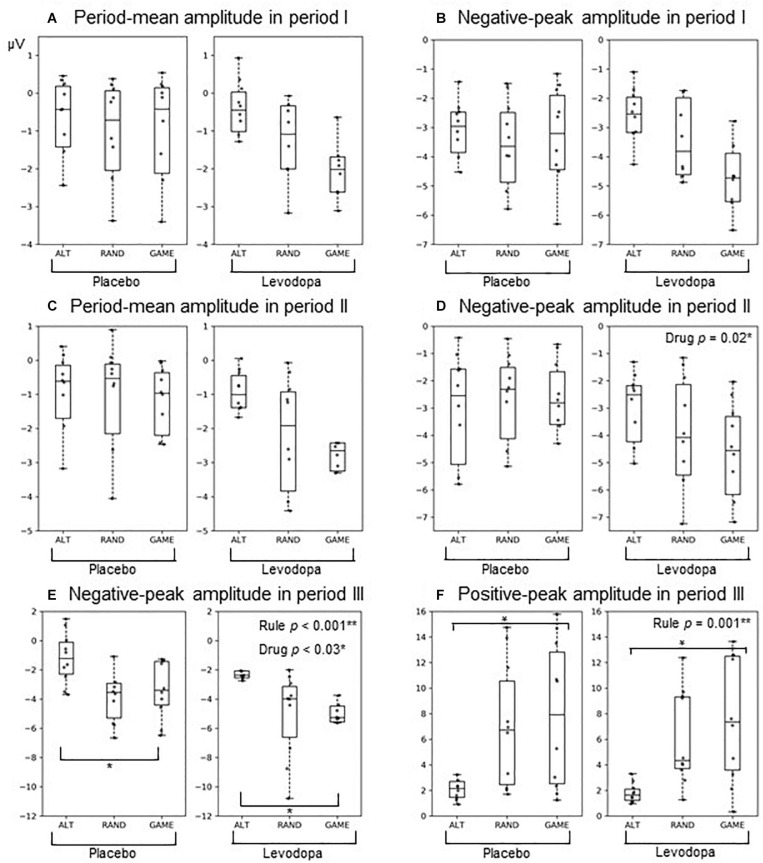
Box plots of ERPs at the Cz electrode: **(A,B)** Period I amplitudes of the period mean **(A)** And negative peak **(B)**. **(C,D)** Period II amplitudes of the period mean **(C)** And negative peak **(D)**. **(E,F)** Period III amplitudes of the negative peak **(E)** And positive peak **(F)**. The format of each box plot is the same as in Figure 2. Dots indicate individual data points.

Figure 6 and Table 3 summarize the amplitudes of ERPs recorded at the Cz electrode during periods I to III and the results of the statistical tests, respectively. The period-mean ERP amplitude in period II showed a tendency of main effects of drug and rule (*p* = 0.06, two-way repeated-measures ANOVA). The negative peak in period II showed a significant main effect of drug (*p* = 0.02). During period III, the ERPs showed negative-to-positive biphasic peaks, and two-way repeated-measures ANOVA was applied to the amplitude of each peak. For the negative peak, there were significant main effects of rule (*p* < 0.001) and drug (*p* = 0.03), while for the positive peak, there was a significant main effect of rule (*p* = 0.001). The grand-averaged waveforms in Figure 4 show that the activity after the subject choice (the time between periods II and III) generally shifted to negative values with levodopa as compared with placebo. When the amplitude of the negative peak in period III was corrected for the baseline amplitude during period II, there was no significant effect of rule or drug (*p* > 0.05, two-way repeated-measures ANOVA).

**TABLE 3:** Results of ERP analysis using two-way repeated-measures ANOVA for rule × drug.

Period	ERP measure	Main effect	*df*	*F*	*p*	η*_p_*^2^	Power
I	Mean	Rule	2	2.01	0.16	0.18	0.36
		Drug	1	0.11	0.75	0.01	0.06
		Interaction	2	1.51	0.25	0.14	0.28
	Negative peak	Rule	2	2.61	0.10	0.01	0.06
		Drug	1	0.09	0.77	0.01	0.05
		Interaction	2	1.97	0.17	0.01	0.06
II	Mean	Rule	2	3.26	0.06	0.27	0.55
		Drug	1	4.59	0.06	0.34	0.48
		Interaction	2	1.31	0.30	0.13	0.25
	Negative peak	Rule	2	1.44	0.26	0.14	0.27
		Drug	1	8.50	0.02*	0.49	0.76
		Interaction	2	2.42	0.12	0.21	0.42
III	Negative peak	Rule	2	18.30	< 0.001**	0.67	1.00
		Drug	1	6.33	0.03*	0.41	0.61
		Interaction	2	0.74	0.49	0.08	0.16
	Positive peak	Rule	2	11.52	0.001**	0.56	0.98
		Drug	1	2.13	0.18	0.19	0.26
		Interaction	2	0.29	0.75	0.03	0.09

df, degrees of freedom. *p < 0.05; **p < 0.01.

**FIGURE 7:**
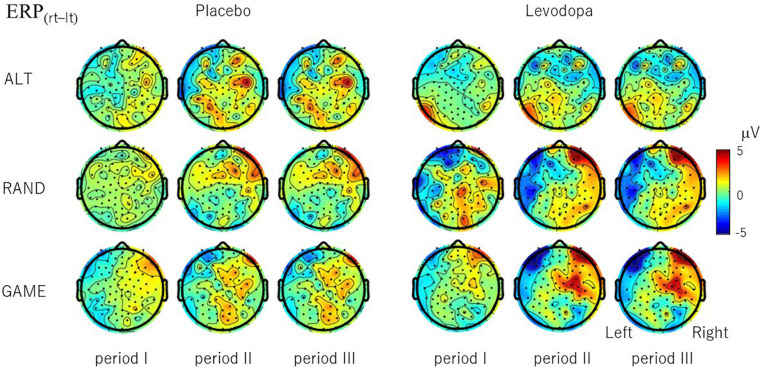
Topographical maps of choice signal ERP_(rt__–__lt)_, which is the difference in the period-mean ERP amplitude between right-choice and left-choice trials. ERP_(rt__–__lt)_ was calculated for each channel and averaged across 10 subjects. For RAND and GAME rules, positive signals evolved in the right hemisphere and negative signals evolved in the left hemisphere during periods II and III with levodopa, but the signal was weaker with placebo.

To examine whether EEG signals differentiate the subject’s choice direction (right–left), we examined the topographical distribution of ERP_(rt__–__lt__)_ (Figure 7). In RAND and GAME sessions with levodopa, clear hemispheric asymmetry emerged after period II, with ERP_(rt__–__lt)_ generally being positive in the right hemisphere and negative in the left hemisphere. The choice signal was not clear in any of the tasks for placebo. Even with levodopa, the signal was absent in ALT sessions. We also examined whether EEG signals differentiated the subject’s choice direction in the previous trial by calculating ERP_(rt__–__lt)_ based on the choice in the preceding trial (Supplementary Material 2). The hemispheric lateralized signal was not present except for in the ALT sessions, in which the previous choice and present choice were contingent on each other.

**FIGURE 8:**
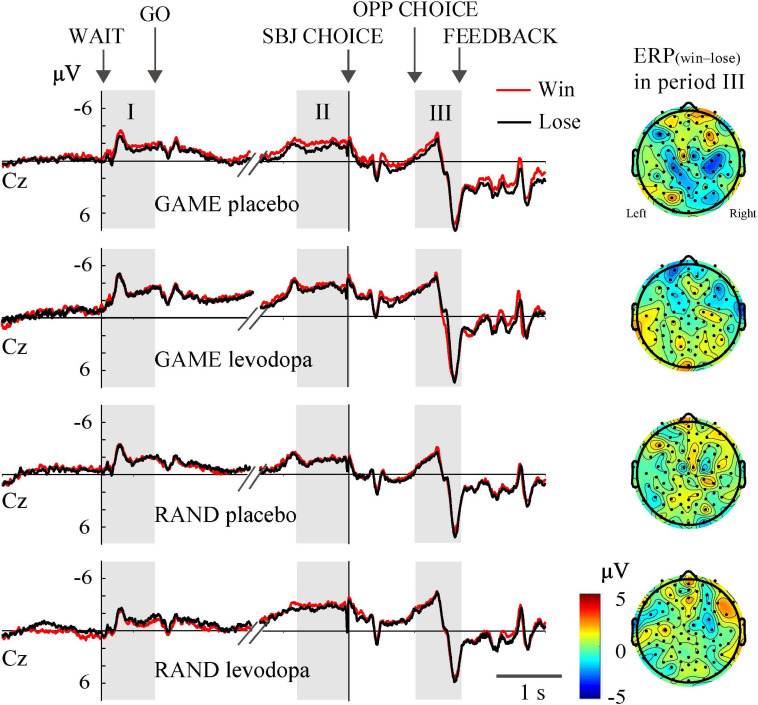
Grand-averaged waveforms at the Cz electrode plotted separately for trials in which the subjects won and lost.

In RAND and GAME sessions, the behavioral winning rates were approximately 0.5. Trials in which the subjects won and lost against the computer player are merged in Figures 4, 5. To examine whether the ERPs differentiated the choice outcome, we plotted the average ERP waveform at the Cz electrode separately for each outcome and examined ERP_(win__–__lose)_ in all recording channels during period III (Figure 8). Three-way ANOVA for rule (ALT, RAND, and GAME) × drug (levodopa and placebo) × outcome (win and lose) revealed that there was no significant main effect of the outcome on the ERPs at the Cz electrode during any periods (*p* > 0.05). Also, ERP_(win__–__lose)_ did not deviate from 0 significantly in any condition (*p* > 0.05, Student’s *t*-test with Bonferroni correction). This meant that the ERPs did not differentiate the decision outcome. We also examined whether the outcome in the previous trial significantly influenced the ERPs by analyzing ERP_p__rev__(win__–__lose)_, which revealed that this parameter did not deviate from 0 in any condition (*p* > 0.05, Student’s *t*-test with Bonferroni correction).

**FIGURE 9:**
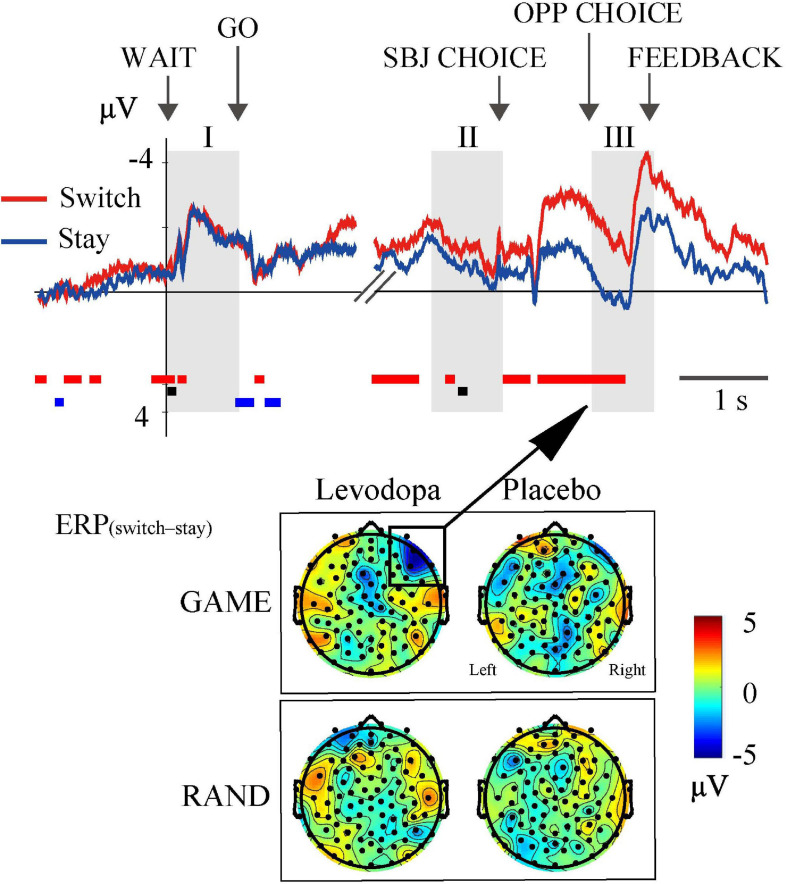
ERP selectivity to switch-or-stay choices. The right lateral frontal area showed negative ERP_(switch__–__stay)_ in GAME with levodopa, indicating that the ERP amplitude was more negative when switching than when staying with the previous target choice. The waveforms are the grand-averaged waveforms in the right lateral frontal area (average of 10 electrodes) in GAME sessions with levodopa. Rectangles below the waveforms indicate time periods where the main effects of choice (red) and drug (black) and the interaction effect were significant [*p* < 0.05; three-way ANOVA for rule × drug × choice (switch and stay)].

To examine whether ERPs reflect the switch-or-stay decision for the direction of target choice, we examined topographical maps of ERP_(switch__–__stay)_ in the GAME and RAND sessions (Figure 9). ERP_(switch__–__stay)_ was negative in the right lateral prefrontal area in GAME sessions with levodopa treatment during period III (top-left topographical map in Figure 9). The ERP selectivity for the switch-or-stay choice was not observed in the RAND sessions (topographical map in the bottom panel of Figure 9). The average ERP in this region in GAME (top waveforms in Figure 9) was significantly more negative on switching (red line) than on staying (blue line) after period II to period III (red rectangles below the waveforms; *p* < 0.05, three-way ANOVA for rule × drug × switch–stay).

## Discussion

We aimed to determine whether ERPs reflect decision strategies when performing a binary choice task. We found that the behavioral performance and reaction time changed systematically according to the decision rule for choosing between right and left targets (Figure 2). Although the subjects made more random choices in GAME than in RAND, the choices were partially predictable, with subjects tending to choose the target on the same side as that previously chosen by the computer player (Figures 2C, 3B). We found that levodopa did not significantly affect the behavioral performance in sessions of any rule. Previous studies that found an influence of levodopa on decision-making were often driven by reward incentives (Chowdhury et al., 2013). Thus, it is possible that cognitive decision-making that does not involve salient reward drive is less sensitive to dopaminergic treatment. In summary, the present behavioral analyses revealed the presence of strategic adaptation in binary decision-making based on the task rule.

We observed sustained negative ERPs after presentation of the WAIT cue. It has been reported that self-paced action induces premovement negative ERPs in the frontocentral region, which is called movement-related potentials (MRPs) (Kornhuber and Deecke, 1965, 2016). The early component of the MRPs is called the Bereitschaftspotential (BP), which is a negative potential that slowly increases a few seconds before executing a movement (Cunnington et al., 1995). There have been several reports of MRP amplitudes being smaller in PD patients than in healthy controls, with the reduced amplitude of the BP being restored by dopaminergic therapy (Amabile et al., 1986; Dick et al., 1987; Feve et al., 1992; Oishi et al., 1995). However, the result was not consistent with another report (Barrett et al., 1986), and the effect of levodopa on MRPs has not been studied in healthy subjects. We found that levodopa enhanced the ERP negativity from period II to period III, indicating the impact of exogenous dopamine on prefrontal activity in healthy human subjects.

Along with the progression of decision-making, ERPs may show selectivity to the chosen direction. To find ERP correlates of the directional decision, we introduced ERP_(rt__–__lt)_ and found that it emerged and developed during the task period. The signal was distributed in the frontocentral region with clear hemispheric laterality (Figure 7). A particularly interesting observation was that the directional signal emerged more slowly in GAME than in RAND, possibly reflecting the difference in decision timing. Importantly, the directional signal was not present in ALT even though the subjects exhibited the same motor responses. These results suggest that the ERPs do not simply encode motor execution, with instead the lateralized directional signal possibly reflecting the cognitive strategy varying according to the task requirements. Another interesting point is that ERP_(rt__–__lt)_ was much weaker without levodopa. Because behavioral measures were not changed significantly by levodopa, the behavioral implication of the levodopa-induced ERP change is unclear. Our speculation is that the prefrontal cortex in healthy subjects has sufficient capacity to perform the task, and so levodopa does not influence behavioral performance due to a ceiling effect. In PD patients whose frontal executive functioning is known to be compromised (Owen, 2004), levodopa treatment may ameliorate the dysfunction and improve the task performance. This hypothesis should be investigated in future studies.

While the present tasks required subjects to choose between right and left targets, it is possible that the decision is made in an alternative form, that is, choosing whether to switch from or stay with the previous choice. We introduced ERP_(switch__–__stay)_ as an indicator of this form of decision-making and found that the ERP correlate was present in the right prefrontal cortex selectively during GAME sessions (Figure 9). Decades of primate neurophysiology research has elucidated the pivotal role of the prefrontal cortex in decision-making. The medial frontal cortex is involved in the voluntary switching of action (Shima and Tanji, 1998; Isoda and Hikosaka, 2007), while the dorsolateral prefrontal cortex (DLPFC) integrates perceptional inputs and reinforcement signals to realize adaptive behavior in a changing environment (Lauwereyns et al., 2001; Sakagami et al., 2001; Gold and Shadlen, 2007; Shadlen and Kiani, 2013). For example, when monkeys forage between two targets following Herrnstein’s matching law, DLPFC neurons encode the valuations of specific choices based on the previous rewards (Tsutsui et al., 2016). Also, when monkeys play a matching task, DLPFC neurons encode their past decisions and reward history (Barraclough et al., 2004). We found that past decisions influenced ERPs in the form of switching from or staying with the previous choice, whereas there was no influence of reward history. The inconsistent results for the same behavioral task are probably attributable to differences in methods. Besides physiological differences between the single-neuron activities and ERPs, the reinforcement method also differed, with the monkeys receiving an immediate liquid reward after their choice and our subjects receiving verbal feedback *via* the monitor as immediate reinforcement.

We observed large biphasic ERPs while the subjects perceived the choice of the opposing player and recognized the behavioral response (period III). These ERPs were prominent in GAME and RAND, but much smaller in ALT (Figure 4). The rule dependency of ERPs may be explained by the information value of the feedback stimulus. The choice of the opposing player was not predictable during RAND and GAME, and so its disclosure represented important information. However, in ALT, the alternating choice of the computer player was perfectly predictable, and disclosing it was not informative. A major finding of the present study is that rule-dependent ERPs were modulated by levodopa administration. This finding is consistent with the role of dopamine in reinforcement learning (Schultz, 2015). Upon receiving the reinforcement signal, the medial prefrontal cortex may compute the action value and update the decision strategy. More specifically, dopaminergic input to the prefrontal cortex might be used to compute the advantages and disadvantages of staying with or shifting the choice in the next trial.

The ERP literature indicates that contingent negative variation (CNV) is elicited in the frontocentral area when two sensory stimuli are paired and presented with a fixed interstimulus interval (Walter, 1964; Tecce, 1972; Rohrbaugh et al., 1976; Hamano et al., 1997). In our study, the intervals between the WAIT and GO cues and between SBJ CHOICE and OPP CHOICE events were fixed. Thus, the ERPs around these events can be conservatively classified as CNV. Whether these ERPs have cognitive components beyond the classical concept of CNV is an important question. To dissociate bottom-up sensory components and top-down cognitive components, researchers have examined how ERPs are modulated by behavioral performance (Gajewski and Falkenstein, 2013; Di Russo et al., 2016, 2017; Perri and Di Russo, 2017). For example, the activity of prefrontal pN and P3 components during a go/no-go task was inversely related to the reaction time variability, which was interpreted as reflecting top-down attention (Perri et al., 2016). Most studies of prefrontal ERPs have used operant tasks, such as the go/no-go task and the Stroop task, in which correct responses are determined by stimulus–response mapping. Thus, in operant tasks, response variability could manifest only in the reaction time and occasional errors. In contrast, the use of a mixed-strategy game in the present study allowed subjects to respond in a nondeterministic manner. We found ERPs that change according to the task rule, choice direction, and decision to switch or stay, and the obtained results provide strong evidence of the influence by top-down executive processes.

The primary factor restricting the generalizability of the present results is the smallness of the sample. Insignificant results in this study may be attributable to an insufficient sample size and the resulting lack of statistical power. For example, it is possible that a larger sample would have revealed significant main effects of rule and drug on the period-mean ERP amplitude during period II. Table 3 provides the power calculations used to estimate the probability of type II errors. The present results should be tested in future research involving a larger number of subjects. The advantages of this study include the relatively large number of trials in each session (Table 2) and its double-blind placebo-controlled design, which are expected to increase the data reliability and statistical power in reducing biases.

In conclusion, we found that prefrontal ERPs reflect different decision strategies when performing a binary choice task. Both the frontomedial negativity and frontolateral signal tuned to the choice direction were modulated by the task rule and levodopa. Important questions to be addressed in future research are how PD patients play mixed-strategy games and whether their ERPs differ from those of healthy subjects.

## Data Availability Statement

The datasets presented in this article are not readily available because the data are still under study for separate analyses. Requests to access the datasets should be directed to SK, skoba-tky@umin.net.

## Ethics Statement

The studies involving human participants were reviewed and approved by the Fukushima Medical University ethical committee. The patients/participants provided their written informed consent to participate in this study.

## Author Contributions

SK designed the study. F-YC, SK, and WW conducted the experiments. YU commented on the experimental data. F-YC and SK analyzed the data and wrote the manuscript. All authors contributed to the article and approved the submitted version.

## Conflict of Interest

The authors declare that the research was conducted in the absence of any commercial or financial relationships that could be construed as a potential conflict of interest.
