# Supplementary material for: Event-Related Potentials During Decision-Making in a Mixed-Strategy Game
Source: Front Neurosci. 2021 Mar 19;15:552750. doi: 10.3389/fnins.2021.552750 (PMC8017162; doi:10.3389/fnins.2021.552750)
Supplement: Supplementary file 1 [file Data_Sheet_1.docx]

5

Influence of the subject’s past choice

0

-5

-10

RAND ON RAND OFF GAME ON

GAME OFF

0 1 2 3 4 5 6 7 8 9 10

N^th^ previous trial

Influence of the opponent’s past choices

5

0

-5

-10

0 1 2 3 4 5 6 7 8 9 10

N^th^ previous trial

Average regression coefficient

Supplementary figure 1

Average regression coefficient

Influence of choices in prior trials evaluated by logistic regression.

Methods

To test how the subject’s choice during random and game tasks was influenced by their own choice and the opposing PC player’s choice, the following logistic regression model was applied using Matlab function ‘mnrfit’:

ln(π_rt_/π_lt_ ) = a + (b1SBJ1 + b2SBJ2 + … +b10SBJ10) + (c1OPP1 + c2OPP2 +… +c10OPP10)

where SBJt and OPPt indicate the choice of the subject and the choice of the opposing PC player in t prior trials (1 for rightward and -1 for leftward; t = 1, 2, …, 10). b_t_ and c_t_ are regression coefficients. The relative log odds of the current choice being right versus left, ln(πrt/πlt), increases exp(bt) and exp(ct) times for each unit increase in SBJt and OPPt, respectively, given all else is equal.

Results

In RAND sessions with levodopa, the subjects tended to choose the side opposite to their choice in the previous trial (b1 = -8.25 ± 15.2; p = 0.1, 2-tailed t-test for difference from 0). Based on the logistic regression model, influences from the past choice of the subjects or opposing PC player in RAND or GAME sessions were not statistically significant.

Different results between the logistic regression method and the log likelihood ratio method (main text and Figure 3) were attributable to poor fitting of the data to the logistic regression, which was reflected in large deviations of fitting. The sums of the deviance residuals were 48.6 ± 18.5, 78.6 ± 178.3, 53.3 ± 14.1, and 86.5 ± 185.7 (mean ± sd), for RAND on, RAND off, GAME on, and GAME off, respectively.
