# Supplementary material for: Event-Related Potentials During Decision-Making in a Mixed-Strategy Game
Source: Front Neurosci. 2021 Mar 19;15:552750. doi: 10.3389/fnins.2021.552750 (PMC8017162; doi:10.3389/fnins.2021.552750)
Supplement: Supplementary file 2 [file Data_Sheet_2.docx]

period II

period III

period III

period I


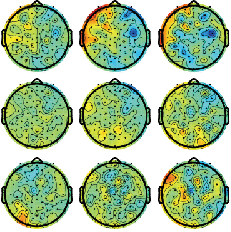

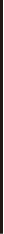

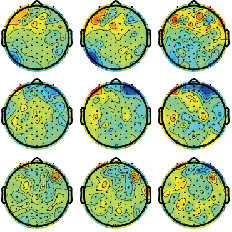

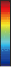


period I

period II

GAME

RAND

ALT

Placebo

Levodopa

μV

-5

0

5

Supplementary figure 2

Influence of subject’s right-left choice in the previous trial on ERPs.

Methods

To test how the subject’s directional choice in the previous trial on the ERPs, we calculated ERP_prev(rt-lt)_.

ERP_prev(rt−lt)_ = ERP_mean prev_(rt) – ERP_mean prev_(lt)

where ERP_mean prev_(rt) and ERP_mean prev_(lt) are the ERPs averaged for trials in which the subject chose the right and left in the previous trials, respectively.

ERP_prev(rt−lt)_ was calculated for each channel for all rule and drug conditions.

Results

Topographical plots are shown above. Compared to the ERP_(rt−lt)_ based on the choice in the current trial (Fig. 7), ERP_prev(rt−lt)_ was smaller and not lateralized.
